# Demographic and Clinical Differences Between Bipolar Disorder Patients With and Without Alcohol Use Disorders

**DOI:** 10.3389/fpsyt.2020.570574

**Published:** 2020-09-03

**Authors:** Yan Xia, Dongying Ma, Tania Perich, Jian Hu, Philip B. Mitchell

**Affiliations:** ^1^ Mental Health Institute, Harbin Medical University, Mental Health Centre, 1st Affiliated Hospital of Harbin Medical University, Harbin, China; ^2^ Department of Neurosurgery, 2nd Affiliated Hospital of Harbin Medical University, Harbin, China; ^3^ School of Psychology, Western Sydney University, Sydney, NSW, Australia; ^4^ School of Psychiatry, University of NSW, Sydney, NSW, Australia

**Keywords:** bipolar disorder, alcohol use disorder, comorbidity, sociodemographics, mental health

## Abstract

**Background:**

Bipolar disorder (BD) and alcohol use disorder (AUD) are two major independent causes of psychopathology in the general population. The prevalence of AUD in BD is high. Identifying the clinical and demographic features of patients with BD who may develop AUD could help with early identification and intervention.

**Methods:**

Data from 238 patients diagnosed with BD were gathered on alcohol use, social demographics, longitudinal course of BD, clinical features of the most severe lifetime manic and depressive episodes, comorbid physical diseases, anxiety disorders, and other substance use disorders.

**Results:**

We found that 74 of 238 BD patients had AUD (67 with alcohol dependence and 7 with alcohol abuse). Bivariate logistic regression analysis and multivariate logistic regression analysis found that the best predictors of AUD in patients with BD were being male (OR = 2.086, 95% CI = 1.094–3.979, *p* = 0.001), younger (OR = 0.965, 95% CI = 0.935–0.996, *p* = 0.026), and comorbidity with other unclassified substance dependence (OR = 10.817, 95% CI = 1.238–94.550, *p* = 0.031).

**Conclusions:**

Male, younger current age, and having other substance use disorders were independently associated with AUD.

## Introduction

Bipolar disorder (BD) and alcohol use disorder (AUD) are two independent major causes of psychopathology in the general population. BD is a mental health disorder that causes periods of depression and periods of abnormally elevated mood (1). It includes bipolar I disorder (which represents classic manic depressive disorder), bipolar II disorder (which is typically characterized by a hypomanic and a depressive episode) and cyclothymic disorder (which is a milder form of bipolar disorder fluctuating between mild depressive and hypomanic symptoms) (2). BD affects 1–4% of the global population (3–5).

AUD is a broad term for any drinking of alcohol that results in mental or physical health problems. The disorder is divided into two types: alcohol abuse and alcohol dependence (6). Alcohol abuse encompasses a spectrum of unhealthy alcohol drinking behaviors, ranging from binge drinking to alcohol dependence. Alcohol dependence is a previous psychiatric diagnosis in which an individual is physically or psychologically dependent upon alcohol. Alcohol abuse has been merged with alcohol dependence into alcohol use disorder in the DSM-5 (6).

AUD is one of the most common comorbid disorders among BD patients (7, 8). AUD is highly prevalent in BD patients with a comorbidity rate ranging from 44 to 62% (3, 9). The comorbidity of AUD in bipolar I disorder (BD I) is higher than in bipolar II disorder (BD II). Lifetime AUD ranges from 46 to 58% in BD I patients, and from 19 to 39% of those with BD II (8, 10). The comorbidity of BD in individuals initially identified with AUD is also high (11).

Several studies have reported that the co-occurrence of AUD and BD is associated with numerous negative consequences including increased severity of manic and depressive symptoms (8, 12, 13), slower recovery from mood episodes, suicide attempts, elevated use of medications and hospitalization, an increased risk for other psychiatric and physical disorders, social dysfunction, and poor quality of life (12–20). A systematic review and meta-analysis showed that comorbid AUD in individuals with BD are significantly associated with suicide attempts (21).

Despite the fact that AUD and BD are associated, the nature of the relationship between these two conditions remains unclear. Previous studies have reported that alcohol has been used to induce pleasurable feelings (22) or to alleviate negative affect and psychiatric symptoms in BD (23). However, Carmiol et al. found that there is a genetic correlation between BD and AUD after statistically controlling for comorbid anxiety, suggesting that genetic factors independent of anxiety influence the risk of BD comorbidity with AUD (24).

The current study aimed to compare a broad range of demographic and clinical features between BD patients with and without comorbid AUD in order to identify the characteristics of patients at a high risk of developing AUD.

## Methods

### Participants

The current study was a cross-sectional design. Between February 2006 and November 2013, a convenience sample of 238 successive patients with BD were recruited from the Depression and Bipolar Clinic of the Black Dog Institute, which is a not-for-profit facility in Sydney, Australia for the diagnosis, treatment, and prevention of mood disorders such as depression and bipolar disorder. Inclusion criteria for BD patients were a BD diagnosis using the DSM-IV and aged between 18 and 85 years old. Participation was voluntary and no eligible patients declined to participate. Patients with comorbid anxiety disorders or other substance abuse or dependence were included. Those with significant neurological or non-psychiatric medical problems were excluded. This study was approved by the Human Research Ethics Committee of University of New South Wales. Written informed consent was obtained from all participants.

### Procedures

The assessments of participants included interviews with questionnaires and ratings by psychiatrists and self-reports completed on a computer.

BD was diagnosed by psychiatrists who had been trained to use the Structured Clinical Interview for DSM-IV-TR disorders (SCID-IV, 2002). Comorbid neurotic-axis (using DSM-IV terminology) disorders and substance abuse disorders over the previous 12 months in patients with BD were assessed using CIDI-AUTO (10, 25). Neurotic-axis disorders assessed included anorexia nervosa, bulimia nervosa, obsessive-compulsive disorder (OCD), post-traumatic stress disorder (PTSD), agoraphobia, social phobia, specific phobia, general anxiety disorder (GAD), and substance abuse disorders included opioids, cannabis, sedatives, cocaine, amphetamines, hallucinogens, inhalants, and PCP.

Six standard scales were used to assess the severity of current symptoms and social functioning: Depression, Anxiety, and Stress Scale (DASS-42) (26); Montgomery-Asberg Depression Scale (MADRS) (27); Bipolar Depression Rating Scale (BDRS) (28); Young Mania Rating Scale (YMRS) (29); Social and Occupational Functioning Assessment Scale (SOFAS) (30); CORE (31) which assesses specific bipolar depression symptoms. Professional psychologists used the face-to-face Composite International Diagnostic Interview (CIDI-AUTO) (32) to get the details of alcohol use including the age of onset of alcohol dependence or abuse and the estimated amount of alcohol per day in last 12 months.

Demographic and general health information was assessed using a self-report questionnaire developed by the investigators including: socio-demographic features (age, gender, country of birth, education, marital status, labor force status, main source of personal income); physical conditions (cerebrovascular, brain trauma, epilepsy, thyroid, cardiovascular, hepatic, renal, hematological, dermatological, skeletal muscular, diabetes). Patients were also interviewed regarding their family history of BD or major depressive disorder, and their number of hospitalizations. The self-report questionnaire was finished by participants on the computer.

### Statistical Analyses

Participants were divided into two groups according to the presence or absence of a comorbid AUD [alcohol abuse (DSM-IV diagnosis code 305.00) or dependence (DSM-IV diagnosis code 303.90)], which was the dependent variable in the current study.

For the independent variables, categorical variables are presented as percentages, and continuous variables are presented as mean±standard deviation (SD). The categorical variables in the demographic and clinical variables between the two groups were compared using chi-square tests. The continuous variables were compared by Mann Whitney-U tests as they were not normally distributed.

As the differences in age and gender between two groups were significant, odds ratios (ORs) and 95% confidence intervals (CI) were determined after adjustment for those two variables for both continuous and categorical comparisons, using binary logistic regression analysis. A stepwise multivariate logistic regression analysis of significant bivariate results was then performed (33). A confidence level of *p* < 0.05 was used for all comparisons. Data analyses were performed using SPSS 22.0 for Windows.

## Results

Of the 238 participants, 74 (31%) had comorbid AUD (67 with alcohol dependence and 7 with alcohol abuse). The mean reported age at onset of AUD was 19 years. The mean reported consumption of alcohol was 7.66 drinks per day in the 12 months prior to assessment, compared to 1.99 drinks per day for the remaining non-AUD participants.

### Features and Potential Risk Factors of Bipolar Patients With and Without Alcohol Use Disorder

In comparisons of the sociodemographic features between two groups, participants with AUD were more likely to be men (*χ^2^* = 3.994, *p* = 0.046); not currently married (*χ^2^* = 7.780, *p* = 0.020); employed (*χ^2^* = 4.781, *P* = 0.029); and younger at enrolment (*U* = 4897.000, *p* = 0.034). For physical health conditions, participants with AUD tended to not have thyroid problems (*χ^2^* = 6.182, *p* = 0.013) and not be allergic to medications (*χ^2^* = 5.667, *p* = 0.017). Additionally, participants with AUD were more likely to: have rapid cycling BD (*χ^2^* = 4.106, *P* = 0.043); report a younger age of their first depressive episode (*U* = 3906.000, *p* = 0.024); more episodes of self-harm (*U* = 808.000, *p* = 0.028); more estimated drinks per day in the last year (*U* = 1589.000, *p* < 0.001); cannabis dependence (*χ^2^* = 9.384, *p* = 0.002); other substance dependence (unclassified, *χ^2^* = 11.915, *p* = 0.001); show symptoms of PTSD (*χ^2^* = 7.545, *p* = 0.006) (**Supplementary Tables 1–4**).

### Logistic Regression Analysis of Potential Risk Factors for Bipolar Patients With Alcohol Use Disorder After Adjusting for Age and Gender

As the differences in age and gender between two groups were significant, they were adjusted in the logistic regression analysis. For sociodemographic features, participants were more likely to be male (OR = 2.256, 95% CI = 1.238–4.111, *p* = 0.008) and separated, divorced, or widowed marital status (OR = 2.657, 95% CI = 1.137–6.206, *p* = 0.024). They were two or more times more likely to inflict self-harm (OR = 3.719, 95% CI = 1.220–11.340, *p* = 0.021), recently report irritability in a past major depressive episode (OR = 2.433, 95% CI = 1.128–5.249, *p* = 0.023) and at a lower age (OR = 0.965, 95% CI = 0.940–0.990, *p* = 0.006) (**Table 1**).

**Table 1:** Logistic regression analysis of potential risk factors in sociodemographic and clinical features of bipolar patients after adjusting for age and gender.

Variables	Without AUD (N = 164)	With AUD (N = 74)	OR (95% CI)	*P* value
	%	%		
Gender (male)	35.0	48.6	2.256 (1.238–4.111)	0.008**
Marital status				
Married or *de facto*	52.2	32.4	2.657 (1.137–6.206)	0.024*
Separated, divorced, or widowed	15.5	21.1	1.785 (0.881–3.618)	0.108
Never married	32.3	46.5		
Labor force status (employed)	47.8	63.4	1.788 (0.982–3.254)	0.057
Education for at least 10 years	89.3	88.2	0.713 (0.279–1.822)	0.479
Main source personal income				
Self employed	44.7	54.9	1.307 (0.506–2.123)	0.922
Spouse or partner’s employment	22.6	12.7	0.672 (0.261–1.734)	0.411
Parental support, student loan	7.5	7.0	1.135 (0.332–3.883)	0.841
Social benefit	25.2	25.4		
Born in Australia	77.8	70.6	0.701 (0.361–1.362)	0.701
Primary diagnosis				
Bipolar I	71.3	63.4	0.442 (0.059–3.300)	0.426
Bipolar II	27.4	33.8	0.587 (0.076–4.536)	0.610
Bipolar disorder due to substance use or general medical condition	0.0	1.4		
Bipolar disorder NOS	1.2	1.4		
Hurt oneself on purpose	46.2	46.4	0.945 (0.520–1.717)	0.853
Number of times patient hurt self on purpose in patients who had attempted suicide				
Once	18.1	7.2	3.719 (1.220–	0.021*
Two or more times	27.1	37.7	11.340)	
Feeling irritable in a past major depressive episode	50.9	74.5	2.433 (1.128–5.249)	0.023*
Family history				
Bipolar disorder	31.0	26.6	0.907 (0.458–1.795)	0.779
Unipolar disorder	56.8	62.5	1.103 (0.582–2.087)	0.764
Hospitalization for BD	69.5	56.5	0.632 (0.343–1.165)	0.141
	Mean ± SD	Mean ± SD		
Age at enrolment	40.556 ± 13.205	36.446 ± 10.750	0.965 (0.940–0.990)	0.006**
Age for first depression episode	21.830 ± 11.075	18.060 ± 8.582	0.974 (0.936–1.013)	0.192
Age for first (hypo)mania episode	25.850 ± 11.210	23.090 ± 10.195	0.992 (0.956–1.029)	0.657
Age for first time to hurt oneself	26.440 ± 25.101	20.780 ± 10.643	0.972 (0.973–2.539)	0.313
Onset age of AUD	NA	18.930 ± 12.655		
Estimated drinks per day last year	1.990 ± 3.416	7.660 ± 5.628		

*Value is statistically significant (p < 0.05) (in bold).

**Value is statistically significant (p < 0.01) (in bold).

NA, not applicable.

In the logistic regression analysis of potential risk factors in comorbidities with substance abuse disorders and anxiety disorders of bipolar patients, cannabis dependence (OR = 5.252, 95% CI = 1.556–17.720, *p* = 0.008), other unclassified substance dependence (OR = 18.900, 95% CI = 2.241–159.381, *p* = 0.007), and PTSD (OR = 2.475, 95% CI = 1.195–5.128, *p* = 0.015) all showed a relationship with the onset of AUD in bipolar patients (**Table 2**).

**Table 2:** Logistic regression analysis of potential risk factors in comorbidities with substance abuse disorders and anxiety disorders of bipolar patients after adjusting for age and gender.

Comorbid diseases	Without AUD (N = 164) (%)	With AUD (N = 74) (%)	OR (95% CI)	*P* value
**Substance abuse disorders**				
Cannabis dependence	2.4	13.5	5.252 (1.556–17.720)	0.008**
Other unclassified substance dependence	0.6	10.8	18.900 (2.241–159.381)	0.007**
Opioid dependence	0.6	1.4	1.905 (0.108–33.670)	0.660
Opioid abuse	0.0	1.4	48,535,353.15 (0.000–)	0.995
Cannabis abuse	1.2	0.0	0.000 (0.000–)	0.997
Sedative dependence	4.3	9.5	12.532 (0.814–7.875)	0.109
Sedative abuse	1.2	2.7	3.208 (0.370–27.813)	0.290
Cocaine dependence	0.6	1.4	1.703 (0.102–28.356)	0.710
Amphetamine dependence	0.6	1.4	2.795 (0.151–51.681)	0.490
Hallucinogen abuse	0.0	1.4	10,730,842.03 (0.000–)	0.995
**Anxiety disorders**				
PTSD	11.6	25.7	2.475 (1.195–5.128)	0.015*
OCD	6.7	12.2	1.607 (0.621–4.157)	0.328
Panic disorder without agoraphobia	4.9	9.5	2.006 (0.676–5.954)	0.210
Panic disorder with agoraphobia	3.7	2.7	0.788 (0.148–4.210)	0.781
Agoraphobia without panic disorder	4.3	1.4	0.350 (0.041–3.005)	0.338
Social phobia	17.1	25.7	1.519 (0.770–2.998)	0.228
Special phobia animal	4.3	6.8	1.604 (0.474–5.426)	0.447
Special phobia natural	3.7	2.7	0.686 (0.131–3.583)	0.655
Special phobia blood	2.4	5.4	1.980 (0.469–8.359)	0.352
Special phobia situation	4.3	2.7	0.562 (0.111–2.840)	0.486

*Value is statistically significant (p < 0.05) (in bold).

**Value is statistically significant (p < 0.01) (in bold).

### Multivariate Logistic Regression Analysis of Potential Risk Factors for Bipolar Patients With Alcohol Use Disorder

The initial stepwise multivariate logistic regression suggested that number of suicide attempts, and irritability in the most severe lifetime depressive episode strongly interfered with other variables due to missing values. So, these two variables were deleted from the final logistic regression model (33).

Multivariate logistic regression analysis of discrepant factors (**Table 3**) demonstrated that being male (OR = 2.086, 95% CI = 1.094–3.979, *p* = 0.001), younger current age (OR = 0.965, 95% CI = 0.935–0.996, *p* = 0.026), and comorbidity with other unclassified substance dependence (OR = 10.817, 95% CI = 1.238–94.550, *p* = 0.031) were most strongly associated with AUD in BD (**Table 3**).

**Table 3:** Multivariate logistic regression analysis of potential risk factors for bipolar patients with alcohol use disorder (AUD).

Variables	OR	95% CI	*P* value
Male	2.086	1.094–3.979	0.026*
Age at enrollment	0.965	0.935–0.996	0.026*
Other unclassified substance dependence	10.817	1.238–94.550	0.031*
Cannabis dependence	3.596	0.960–13.478	0.058
PTSD	1.700	0.744–3.887	0.208
Separated, divorced, or widowed	2.245	0.922–5.466	0.075

*Value is statistically significant (p < 0.05).

## Discussion

This study found that 74 (31%) of 238 BD patients had comorbid AUD. AUD participants were more likely to be single men who had harmed themselves more often, had their first depressive episode younger, experienced irritability during their most severe depressive episode, reported substance dependence additionally on something other than alcohol, and reported PTSD.

Many international studies have reported high rates of comorbidity between AUD and BD. Similar rates of BD with comorbid AUD have been reported in recent epidemiological surveys in Australian communities (27 and 32% respectively reported by Mitchell et al. (5) and Morgan et al. (34).

Here, male BD patients had a 2.26 times greater risk of comorbid AUD than female BD patients, which is consistent with several other studies (7, 8, 12–14, 33, 35). A meta-analysis of clinical studies found that being male was associated with double the lifetime prevalence of AUDs in BD (44%), compared to being female (22%) (35). As in the present study, male BD patients had 2–3 times greater risk of lifetime AUD. Frye et al. found that more men with BD met the criteria for lifetime alcoholism in the general population. However, compared to general population rates, Frye et al. found women with BD (OR = 7.35) have a higher risk of comorbid AUD than for men with BD (OR = 2.77) (11).

We found that those BD patients with comorbid AUD were 2.66 times more likely to be divorced, separated, or widowed. This finding is consistent with results from the US National Epidemiologic Survey on Alcohol and Related Conditions, which reported that rates of substance abuse and dependence were generally greater among men, native Americans, respondents aged 18 to 44 years, those of lower socioeconomic status, those residing in the Western United States, and those who were never married or were widowed, separated, or divorced (14). Here, levels of educational attainment or income source (i.e., personal income or government social support) did not differ between groups.

The prevalence of BD subgroups (particularly BD-I and BD-II) did not differ between the two groups in this study, a finding inconsistent with some previous reports. Several studies have reported a higher prevalence of AUD in patients with BD-I compared to BD-II disorder (Chengappa et al: 49.3 *vs*. 38.9% (36), Frye et al: 41.1 *vs*. 25.0% (11), Regier et al: 46.2 *vs*. 39.2% (37)). But the prevalence of AUDs was higher in BD-II disorder than in BD-I (26.8 *vs*. 14.9%) in Simhandl’s research (8). It is possible that this lack of difference may have been due to the relatively low proportion of BD-II patients in this sample (only 31% had BD-II).

For those BD patients with comorbid AUD who had attempted suicide, the number of reported self-harming events was higher than those without AUD who had also attempted suicide. Chen and Dilsaver reported that 25–60% of BD patients had at least one suicide attempt (38). Cardoso et al. found that BD patients with AUD were more likely to have attempted suicide and a trend that the more severe the alcohol comorbidity, the higher the number of suicide attempts (13). A more recent Brazilian study has reported that more BD patients with AUD had at least one suicide attempt and had also attempted suicide more frequently (33). This finding is also consistent with results from the US National Epidemiologic Survey on Alcohol and Related Conditions (9).

There were more patients with AUD who reported irritability in their most severe lifetime depressive episode. This is a novel finding, which has not been previously reported. This observation may be related to the common causal factors related to the genetics of AUD and BD. And patients are prone to heavier drinking when they are depressed. The irritability is a common result of hangovers.

We did not find any differences in the age of first onset of mood episodes, number of lifetime episodes, likelihood of psychotic symptoms, or rates of hospitalization in the AUD group. This is not consistent with previous studies, which have tended to show a worse prognosis in BD patients with AUD (8, 12, 13, 33, 39, 40). Those studies have reported that individuals with comorbid BD and substance-use disorders have a more severe course of illness, including earlier onset, more frequent episodes, and more complications, including anxiety- and stress-related disorders, aggressive behavior, legal problems, and suicide (40). Compared to the present study, these studies tend to have used longer data collection periods. It has been reported that BD patients with AUD have more psychiatric hospitalizations after adjusting for gender, race, employment status, education level, and duration of illness (41). The study from Simhandl et al. suggested that comorbid AUD had a negative impact on bipolar I participants, increasing depressive episodes (8). A recent study also confirmed the role of the male gender, additional substance use disorder, and irritable and hyperthymic temperaments as predictors of AUD in BD (12).

There were higher rates of rapid cycling in the AUD group, which was not significant using a conservative approach of controlling for age and gender. There were no significant differences in the severity of cross-sectional symptoms. Other groups have reported that BD patients with AUD have higher rates of mixed or dysphoric mania, rapid cycling, increased severity of manic and depressive symptoms (42, 43), and higher levels of novelty seeking, suicidality, aggressiveness, and impulsivity (14). Cardoso et al. found that BD patients with AUD showed more depressive symptoms and poorer functioning, as indicated by higher Hamilton Depression Rating Scale scores and lower scores on the Global Assessment of Functioning Scale (13). Other groups have also reported rapid cycling to be associated with AUD in BD (33, 39, 44, 45). Cruz et al. found that a greater percentage of BD patients with rapid cycling had a history of AUD than those without rapid cycling (44). BD patients with AUD were more likely to present with rapid cycling in a Brazilian sample (33). These studies had larger sample sizes than the present study, so may have been more sensitive to detect small effects.

We found higher rates of comorbidity with PTSD, cannabis dependence, and other substance dependence in those with AUD. These findings are consistent with a number of prior reports (7, 13, 33, 46–48). Nery et al. found that BD patients with AUD had a significantly higher prevalence of comorbid substance (non-alcohol) use disorders, PTSD, and anorexia (33). More than 50% of patients with co-occurring BD and alcohol dependence were diagnosed with at least one anxiety (76.7%) or drug dependence disorder (60.0%) in the study by Prisciandaro et al. Comorbid anxiety disorders were prospectively associated with increased depressive symptoms and alcohol use (48).

Here, multivariate logistic regression showed that male, younger age, and with other unclassified substance dependence (DSM-IV diagnosis code 304.90) were strongly related to AUD with BD. Few other studies of AUD in BD have reported the findings of multivariate logistic regression analyses. This finding is partly consistent with results from one of the few other studies (33). In this study, variables including male gender, younger age at onset of the mood disorder, a lifetime substance (non-alcohol) use disorder, lifetime PTSD diagnosis, history of lifetime suicide attempts, and a family history of substance use disorders were most strongly associated with a lifetime AUD diagnosis in BD patients. Drinking and depression appeared to have a positive relationship among men (49).

### Limitations

First, the study features a relatively small sample size. More participants will be recruited in a future study. Second, the study was cross-sectional, so the findings can only demonstrate associations rather than directional causality. Third, participants were all recruited from a single clinic. The results of this study cannot necessarily be extrapolated to people from other geographical locations or other types of service. Fourth, the present study did not determine whether BD manifested before AUD or vice versa and the CIDI-AUTO’s measures of alcohol intake is relatively crude. Some form of diary or quantity-frequency measure would be preferable. Fifth, there was no comparison of BD I *versus* BD II in the current analysis due to lack of data (or concern of the lack of power in the subgroup analysis). Sixth, the ethnicity of the participants was not investigated in this study, therefore it may not be possible to extend the results to people from diverse ethnic backgrounds. Seventh, data on medications was not gathered, although medication can affect brain structure (50), behavior (51), and substance abuse (52), which will be explored in the future study.

## Conclusions

This study found that being male, younger, and having another unclassified substance dependence were strongly related to AUD in BD. In the BD clinic, younger single men who have self-harmed more than once and are known to be polysubstance users may be at higher risk of AUD. This knowledge could help early identification and intervention.

## Data Availability Statement

The original contributions presented in the study are included in the article/**Supplementary Materials**; further inquiries can be directed to the corresponding authors.

## Ethics Statement

The studies involving human participants were reviewed and approved. This study was approved by the Human Research Ethics Committee of University of New South Wales. All procedures performed in studies involving human participants were in accordance with the ethics standards of the institutional and national research committee and with the 1964 Helsinki Declaration and its later amendments or comparable ethics standards. The patients/participants provided their written informed consent to participate in this study.

## Author Contributions

YX, DM, JH and PM conceived and designed research. YX and DM collected data and conducted research. YX, DM, TP, JH and PM analyzed and interpreted data. YX and DM wrote the initial paper. TP, JH and PM revised the paper. JH had primary responsibility for final content. All authors contributed to the article and approved the submitted version.

## Funding 

This research was supported by the National Natural Science Foundation of China (No. 81501147) to YX and the National Natural Science Foundation of China (No. 61773134) to DM. The study was also supported by the Australian National Medical and Health Research Council (Program Grant 1037196). The authors declare that they have no financial relationship with the organization that sponsored the research, and the funding body was not involved in study design, data collection, analysis and writing of the study. This research was also supported by the National Key R&D Program of China (No. 2018YFC1314400, 2018YFC1314402) to JH.

## Conflict of Interest

The authors declare that the research was conducted in the absence of any commercial or financial relationships that could be construed as a potential conflict of interest.
