# Supplementary material for: Demographic and Clinical Differences Between Bipolar Disorder Patients With and Without Alcohol Use Disorders
Source: Front Psychiatry. 2020 Sep 3;11:570574. doi: 10.3389/fpsyt.2020.570574 (PMC7495181; doi:10.3389/fpsyt.2020.570574)
Supplement: Supplementary file 1 [file Table_1.doc]

**Supplementary Table 1.** Sociodemographic features of bipolar patients with and without AUD

| Variables | BD patients without AUD  (N = 164) | BD patients with AUD  (N = 74) | Statistical test | *P* value |
| --- | --- | --- | --- | --- |
|  | % | % |  |  |
| Gender  Male  Female | 35.0  65.0 | 48.6  51.4 | *χ^2^* = 3.994  *df* = 1 | 0.046* |
| Marital status  Married or de facto  Separated, divorced, or widowed  Never married | 52.2  15.5  32.3 | 32.4  21.1  46.5 | *χ^2^* = 7.780  *df* = 2 | 0.020* |
| Labor force status  Employed  Unemployed | 47.8  52.2 | 63.4  36.6 | *χ^2^* = 4.781  *df* = 1 | 0.029* |
| Education for at least 10 years | 89.3 | 88.2 | *χ^2^* = 0.056  *df* = 1 | 0.813 |
| Main source personal income  Self employment  Spouse or partner’s employment  Parental support, student loan  Social benefit | 44.7  22.6  7.5  25.2 | 54.9  12.7  7.0  25.4 | *χ ^2^* = 3.593  *df* = 3 | 0.309 |
| Born in Australia | 77.8 | 70.6 | *χ^2^* = 1.344  *df* = 1 | 0.246 |
|  | Mean ± SD | Mean ± SD |  |  |
| Age at enrolment | 40.556 ± 13.205 | 36.446 ± 10.750 | *U* = 4897.000 | 0.034* |
| Months since last employment | 30.390 ± 93.532 | 6.560 ± 26.937 | *U* = 5073.000 | 0.082 |

* Value is statistically significant (*p* < 0.05).

**Supplement Table 2.** Physical health conditions of bipolar patients with and without AUD

| Physical diseases | BD patients without AUD  (N = 164) (%) | BD patients with AUD  (N = 74) (%) | *χ^2^*  (*df* = 1) | *P* value |
| --- | --- | --- | --- | --- |
| Heart disease | 5.1 | 5.6 | 0.000 | 1.000 |
| Peripheral-vascular problems | 5.6 | 7.1 | 0.021 | 0.886 |
| Thyroid problems | 18.2 | 5.7 | 6.182 | 0.013* |
| Allergy to medication or tablets | 40.6 | 24.3 | 5.667 | 0.017* |
| Illness requiring treatments with cortisone or prednisone | 17.5 | 22.9 | 0.880 | 0.348 |
| High blood pressure | 9.4 | 10.0 | 0.018 | 0.893 |
| Anaemia | 15.2 | 10.1 | 1.037 | 0.309 |
| High cholesterol or triglycerides | 12.0 | 8.5 | 0.644 | 0.422 |
| Diabetes or sugar problem | 8.9 | 9.9 | 0.059 | 0.809 |
| Liver disease or jaundice | 5.7 | 8.6 | 0.281 | 0.596 |

| Kidney problem | 3.8 | 5.6 | 0.089 | 0.765 |
| --- | --- | --- | --- | --- |

| Severe skin disease | 11.9 | 12.9 | 0.044 | 0.834 |
| --- | --- | --- | --- | --- |
| Muscular problems | 15.1 | 14.3 | 0.025 | 0.874 |
| Rheumatoid arthritis | 6.3 | 4.2 | 0.101 | 0.751 |

| Non-rheumatoid arthritis or joint problems | 22.0 | 17.1 | 0.707 | 0.401 |
| --- | --- | --- | --- | --- |

| Meningitis or encephalitis | 2.5 | 2.8 | 0.000 | 1.000 |
| --- | --- | --- | --- | --- |

| Migraine | 9.3 | 15.3 | 1.783 | 0.182 |
| --- | --- | --- | --- | --- |

| Head injury | 16.5 | 18.6 | 0.153 | 0.696 |
| --- | --- | --- | --- | --- |

| Epilepsy, fits, funny turns | 8.9 | 12.7 | 0.789 | 0.374 |
| --- | --- | --- | --- | --- |

| Glaucoma or eye disease | 1.9 | 4.2 | 0.346 | 0.557 |
| --- | --- | --- | --- | --- |

| Cancer or tumour | 7.5 | 5.6 | 0.055 | 0.815 |
| --- | --- | --- | --- | --- |

| Severe infection in the past 3 years | 6.3 | 9.9 | 0.940 | 0.332 |
| --- | --- | --- | --- | --- |

| Any other significant medical illness required treatment | 36.7 | 31.0 | 0.706 | 0.401 |
| --- | --- | --- | --- | --- |

* Value is statistically significant *(p* < 0.05).

**Supplementary Table 3.** Categorical variables of BD in bipolar patients with and without AUD.

| Variables | | BD patients without AUD  (N = 164) (%) | | BD patients with AUD  (N = 74) (%) | | Statistical test | | *P* value |  |
| --- | --- | --- | --- | --- | --- | --- | --- | --- | --- |
|  | | % | | % | |  | |  |  |
| Primary diagnosis  Bipolar I  Bipolar II  Bipolar disorder due to substance use or general medical condition  Bipolar disorder NOS | | 71.3  27.4  0.0  1.2 | | 63.4  33.8  1.4  1.4 | | *χ^2^* = 3.484  *df* = 3 | | 0.323 |  |
| Pattern of BD  (Hypo)mania/ depression/ recovery  Depression/ (hypo)mania/ recovery  Periods of recovery or remission between each episode of (hypo)mania or depression  Continual cycling between episodes  No clear pattern | | 36.2  13.2  7.2  16.4  27.0 | | 47.1  12.8  4.3  12.9  22.9 | | *χ^2^* = 2.862  *df* = 4 | | 0.581 |  |
| Seasonal pattern associated with (hypo)mania episodes | | 22.9 | | 25.4 | | *χ^2^* = 0.149  *df* = 1 | | 0.699 |  |
| Seasonal pattern associated with depression episodes | | 30.3 | | 32.3 | | *χ^2^* = 0.074  *df* = 1 | | 0.785 |  |
| Rapid cycling | | 59.3 | | 73.8 | | *χ^2^* = 4.106  *df* = 1 | | 0.043* |  |
| Family history of bipolar disorder | | 31.0 | | 26.6 | | *χ^2^* = 0.426  *df* = 1 | | 0.514 |  |
| Family history of unipolar disorder | | 56.8 | | 62.5 | | *χ^2^* = 0.580  *df* = 1 | | 0.446 |  |
| Hospitalization for BD | | 69.5 | | 56.5 | | *χ^2^* = 3.540  *df* = 1 | | 0.060 |  |
| Involuntary bipolar admissions | | 51.9 | | 46.2 | | *χ^2^* = 0.378  *df* = 1 | | 0.539 |  |
| Hurt oneself on purpose | | 46.2 | | 46.4 | | *χ^2^* = 0.001  *df* = 1 | | 0.975 |  |
| When self-harmed  Wanted to die  Ambivalent about dying | | 37.7  49.3 | | 32.3  48.4 | | *χ^2^* = 0.725  *df* =2 | | 0.696 |  |
|  | Mean ± SD | | Mean ± SD | |  | |  | | |
| BMI | 27.564 ± 5.607 | | 26.892 ± 6.514 | | *U* = 3840.500 | | 0.419 | | |
| MADRS | 14.405 ± 12.877 | | 14.296 ± 10.578 | | *U* = 5359.000 | | 0.589 | | |
| BDRS | 13.428 ± 10.801 | | 14.708 ± 9.890 | | *U* = 4232.500 | | 0.240 | | |
| YMRS | 3.363 ± 4.677 | | 4.478 ± 4.904 | | *U* = 4594.000 | | 0.065 | | |
| SOFAS | 65.850 ± 16.636 | | 66.83 ± 15.135 | | *U* = 4592.500 | | 0.531 | | |
| DASS-42 | 47.228 ± 30.782 | | 55.409 ± 27.472 | | *U* = 3995.500 | | 0.055 | | |
| DASS-depression | 18.309 ± 11.084 | | 20.797 ± 9.282 | | *U* = 4365.000 | | 0.073 | | |
| DASS-anxiety | 14.884 ± 9.733 | | 16.838 ± 8.937 | | *U* = 4314.500 | | 0.123 | | |
| DASS-stress | 16.601 ± 10.620 | | 17.985 ± 10.093 | | *U* = 4441.500 | | 0.310 | | |
| CORE | 5.893 ± 5.014 | | 4.824 ± 5.330 | | *U* = 323.000 | | 0.241 | | |
| CORE-non-interactiveness | 3.000 ± 2.394 | | 3.111 ± 2.742 | | *U* = 166.500 | | 0.905 | | |
| CORE-retardation | 3.629 ± 2.713 | | 3.308 ± 3.011 | | *U* = 199.000 | | 0.499 | | |
| CORE-agitation | 1.895 ± 1.370 | | 1.375 ± 0.518 | | *U* = 65.000 | | 0.585 | | |
| Number of admission for (hypo)mania | 1.080 ± 1.571 | | 1.640 ± 2.147 | | *U* = 1459.500 | | 0.180 | | |
| Number of admission for depression | 2.310 ± 3.034 | | 2.470 ± 3.210 | | *U* = 1647.500 | | 0.870 | | |
| Age for first (hypo)mania episode | 25.850 ± 11.210 | | 23.090 ± 10.195 | | *U* = 4071.500 | | 0.064 | | |
| Number of (hypo)mania episodes | 18.550 ± 32.990 | | 21.480 ± 32.841 | | *U* = 2476.000 | | 0.232 | | |
| Age for first depression episode | 21.830 ± 11.075 | | 18.060 ± 8.582 | | *U* = 3906.000 | | 0.024* | | |
| Number of depression episodes | 23.930 ± 49.986 | | 17.480 ± 24.467 | | *U* = 1957.000 | | 0.842 | | |
| Number of depression episodes before first experienced (hypo)mania | 1.710 ± 2.655 | | 1.980 ± 3.363 | | *U* = 2259.000 | | 0.476 | | |
| Age at first purposeful self- harm | 26.440 ± 25.101 | | 20.780 ± 10.643 | | *U* = 854.500 | | 0.055 | | |
| Number of times purposeful self-harm | 1.857 ± 0.889 | | 2.290 ± 0.973 | | *U* = 808.000 | | 0.028* | | |
| Times intended to die when self-harmed | 2.570±2.188 | | 2.430 ± 1.830 | | *U* = 464.000 | | 0.788 | | |
| Times ambivalent about living or dying when self-harmed | 1.000±1.606 | | 0.460 ± 0.967 | | *U* = 162.000 | | 0.191 | | |
| Onset age of AUD | NA | | 18.930 ± 12.655 | |  | |  | | |
| Estimated drinks per day last year | 1.990±3.416 | | 7.660±5.628 | | *U* = 1589.000 | | <0.001** | | |

NA: Not applicable

* Value is statistically significant (*p* < 0.05).

** Value is statistically significant (*p* < 0.01).

**Supplementary Table 4.** Comorbidities with substance abuse disorders and anxiety disorders in bipolar patients with and without AUD

| Comorbid diseases | BD patients without AUD  (N = 164) (%) | BD patients with AUD  (N = 74) (%) | *χ^2^*  (*df* = 1) | *P* value |
| --- | --- | --- | --- | --- |
| **Substance abuse disorders** |  |  |  |  |
| Opioid dependence | 0.6 | 1.4 | 0.000 | 1.000 |
| Opioid abuse | 0.0 | 1.4 | 0.168 | 0.682 |
| Cannabis dependence | 2.4 | 13.5 | 9.384 | 0.002** |
| Cannabis abuse | 1.2 | 0.0 | 0.035 | 0.852 |
| Sedative dependence | 4.3 | 9.5 | 1.633 | 0.201 |
| Sedative abuse | 1.2 | 2.7 | 0.078 | 0.780 |
| Cocaine dependence | 0.6 | 1.4 | 0.000 | 1.000 |
| Amphetamine dependence | 0.6 | 1.4 | 0.000 | 1.000 |
| Hallucinogen abuse | 0.0 | 1.4 | 0.168 | 0.682 |
| Other unclassified substance dependence | 0.6 | 10.8 | 11.915 | 0.001** |
|  |  |  |  |  |
| **Anxiety disorders** |  |  |  |  |
| OCD | 6.7 | 12.2 | 1.971 | 0.160 |
| PTSD | 11.6 | 25.7 | 7.545 | 0.006** |
| Panic disorder without agoraphobia | 4.9 | 9.5 | 1.120 | 0.290 |

| Panic disorder with agoraphobia | 3.7 | 2.7 | 0.000 | 1.000 |
| --- | --- | --- | --- | --- |

| Agoraphobia without panic disorder | 4.3 | 1.4 | 0.589 | 0.443 |
| --- | --- | --- | --- | --- |
| Social phobia | 17.1 | 25.7 | 2.381 | 0.123 |
| Special phobia animal | 4.3 | 6.8 | 0.242 | 0.623 |

| Special phobia natural | 3.7 | 2.7 | 0.000 | 1.000 |
| --- | --- | --- | --- | --- |

| Special phobia blood | 2.4 | 5.4 | 0.619 | 0.431 |
| --- | --- | --- | --- | --- |

| Special phobia situation | 4.3 | 2.7 | 0.048 | 0.827 |
| --- | --- | --- | --- | --- |

** Value is statistically significant (*p* < 0.01).
